# NRVS Spectroscopy Resolves Distinct Bridging Hydride Intermediates in [NiFe]-Hydrogenase

**DOI:** 10.1021/jacs.5c15408

**Published:** 2025-10-31

**Authors:** Giorgio Caserta, Konstantin Laun, Jean-Pierre Oudsen, Ilya Sergueev, Ingo Zebger, Oliver Lenz

**Affiliations:** † Institut für Chemie, 26524 Technische Universität Berlin, Straße des 17. Juni 135, 10623 Berlin, Germany; ‡ Deutsches Elektronen-Synchrotron, Notkestraße 85, 22607 Hamburg, Germany

## Abstract

The active-site iron of an H_2_-sensing [NiFe]-hydrogenase was selectively labeled with ^57^Fe, allowing the probing of all catalytic intermediates using synchrotron-based nuclear resonance vibrational spectroscopy. Diagnostic metal–hydride vibrations were detected for both the Ni_a_-C and Ni_a_-SR intermediates, with their assignments being confirmed through H/D isotope substitution and in situ hydride photolysis experiments. Interestingly, these Fe–hydride bands are separated by a large energy gap, reflecting distinct bonding interactions at the metal–hydride site. Despite these differences, vibrational analyses across all catalytically active species reveal a conserved structural rigidity of the [NiFe] center, which appears crucial for sustaining efficient and rapid electron transfer in [NiFe]-hydrogenases.

## Graphical abstract

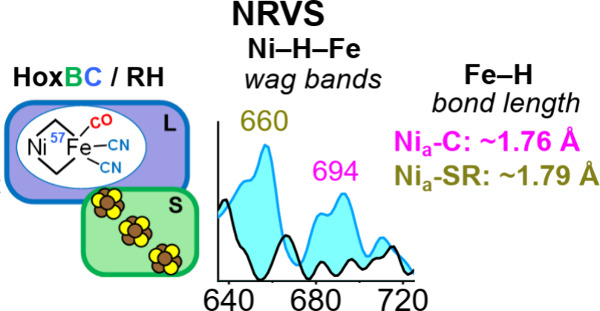

[NiFe]-hydrogenases are multisubunit metalloenzymes containing a minimal bipartite (large and small subunits) catalytic unit to split or produce H_2_.^1,2^ Catalysis takes place at a heterobimetallic NiFe­(CN)_2_CO bioinorganic cofactor that is covalently bound to the large subunit by four cysteine residues.^3,4^ Electrons are shuttled to redox partners via a relay of iron–sulfur clusters located in the small subunit, while protons are transferred via dedicated proton transfer pathway(s).^5,6^ H_2_ conversion involves a series of active site intermediates, and despite the efforts of various research groups, their exact number and structure is still debated.^7–10^ Spectroscopic methods are at the forefront of hydrogenase research, whereby infrared (IR) spectroscopy is used to study the redox and structure sensitive vibrational bands originating from the active site CO and CN^–^ ligands, and electron paramagnetic resonance (EPR) spectroscopy is used to study unpaired electrons from the inorganic iron–sulfur clusters and [NiFe] center and certain organic (e.g., FAD, FMN) cofactors.^7,11,12^ However, both techniques are limited in their ability to precisely characterize the structures of [NiFe]-hydrogenase catalytic intermediates (Figure S1). Over the past decade, resonance Raman (rR) spectroscopy has also provided valuable insights into metal–ligand and intra–ligand vibrations at the bimetallic active site of [NiFe]-hydrogenases.^13,14^ Nonetheless, the required high laser power densities have so far prevented the rR spectroscopic characterization of the Ni_a_-SR and Ni_a_-C species, whose bridging hydrides are photolabile. Synchrotron-based nuclear resonance vibrational spectroscopy (NRVS) provided further valuable insights into catalytic and biosynthetic hydrogenase intermediates by allowing selective detection of vibrational modes associated with Mössbauer-active nuclei such as ^57^Fe.^3,15–17^ [NiFe]-hydrogenases contain many iron ions, most of them in the form of multiple iron–sulfur clusters. Thus, NRVS data of uniformly ^57^Fe-labeled enzymes displays predominantly contributions from Fe–S­(−Fe) stretching and bending modes in the region between 100 and 420 cm^–1^.^18^ Vibrational bands associated with the single iron at the catalytic site, including those of hydride-containing intermediates, are confined to the 420–700 cm^–1^ region, dominated by Fe–CO/CN stretching and bending modes.^19^ Detecting Fe/Ni–H vibrations, however, remains particularly challenging owing to their intrinsically low intensity.^20,21^ In 2015 Cramer, Lubitz and co-workers reported on NRVS data for one Ni_a_-SR subform using uniformly ^57^Fe-labeled *Desulfovibrio vulgaris* Miyazaki F (*Dv*MF) [NiFe]-hydrogenase,^22^ where they could unambiguously assign an H/D-sensitive Ni–H–Fe wagging vibration. In contrast, a recently reported NRVS characterization of the Ni_a_-C intermediate using highly concentrated, lyophilized regulatory [NiFe]-hydrogenase from *Cupriavidus necator* (*Cn*RH) lacked the expected Ni–H–Fe wagging vibration calculated by DFT.^23^ This raised the question of whether such a small spectral feature is detectable for the Ni_a_-C intermediate. Recently, we developed a biochemical procedure that enables the *in vitro* assembly of a fully functional *Cn*RH based on independently isolated large (HoxC) and small (HoxB) subunits.^24^ Using this procedure, we achieved unprecedentedly selective and complete ^57^Fe-labeling of the [NiFe] site, and we demonstrated that it is applicable to record NRVS data of the H_2_ binding Ni_a_-S intermediate. In the current study, we further exploited the selective labeling of the active site iron to characterize the complete set of Fe–ligand vibrations across all known catalytic intermediates.

The ^57^Fe-labeled catalytic subunit HoxC of *Cn*RH and the iron–sulfur cluster-containing HoxB subunit were purified and assembled as described before.^24,25^ In line with previous IR spectroscopic investigations, the *in vitro* assembled HoxBC complex was virtually indistinguishable from native *Cn*RH and displayed the characteristic absorptions related to the CO and CN^–^ ligands of the Ni_a_-C (ν_CO_= 1961 cm^–1^) and Ni_a_-SR (ν_CO_ = 1948 cm^–1^) intermediates in the reduced enzyme and the Ni_a_-S intermediate (ν_CO_ = 1943 cm^–1^) in the reoxidized enzyme (Figure S2). An H_2_-reduced HoxBC sample was then concentrated to ∼1.2 mM and additionally incubated with sodium dithionite to prevent reoxidation. IR spectroscopic analysis (Figure 1) of the H_2_/dithionite-reduced HoxBC sample (HoxBC_red_) revealed an increased population of the Ni_a_-SR intermediate and its subform Ni_a_-SR′ (ν_CO_ = 1935 cm^–1^). Integration of the CO bands revealed that ∼60% of active site was present in the Ni_a_-C intermediate, while the remaining 40% were distributed between the two Ni_a_-SR subforms. Figure 2a shows the NRVS spectrum of HoxBC_red_ compared to previously published data on uniformly ^57^Fe-labeled *Dv*MF hydrogenase enriched in the Ni_a_-SR intermediate.^22^ The selective ^57^Fe-labeling of the HoxBC active site resulted in a remarkable increase in the relative intensity of the Fe–CO/CN-derived bands in the range of 400–620 cm^–1^. Analysis of the HoxBC_red_ spectrum revealed intense bands at 545 and 555, 600, and 614 cm^–1^, which are mainly associated with Fe–CO vibrational modes, and a weaker band at 578 cm^–1^.

**Figure 1:**
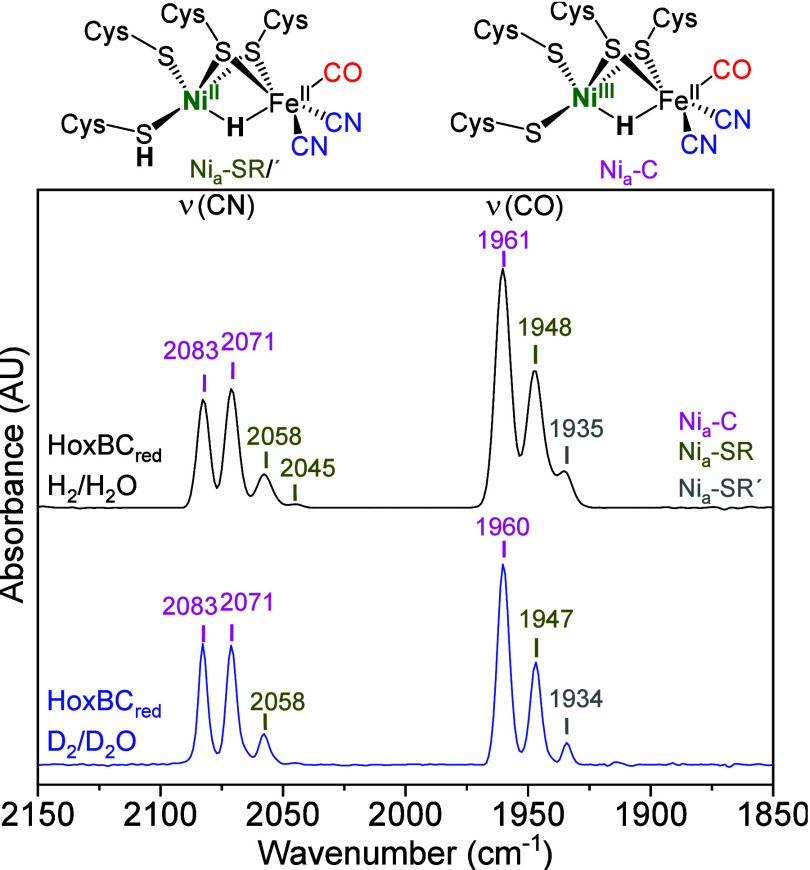
IR spectra of reduced HoxBC with absorptions related to the stretching vibrations of the CO and CN^–^ ligands of the [NiFe]-hydrogenase active site. Black: HoxBC_red_ in H_2_/H_2_O; blue: HoxBC_red_ in D_2_/D_2_O. Color code: Ni_a_-C, magenta; Ni_a_-SR, olive yellow; Ni_a_-SR′, gray. Sketches of the Ni_a_-C and Ni_a_-SR intermediates are shown on the top.

**Figure 2:**
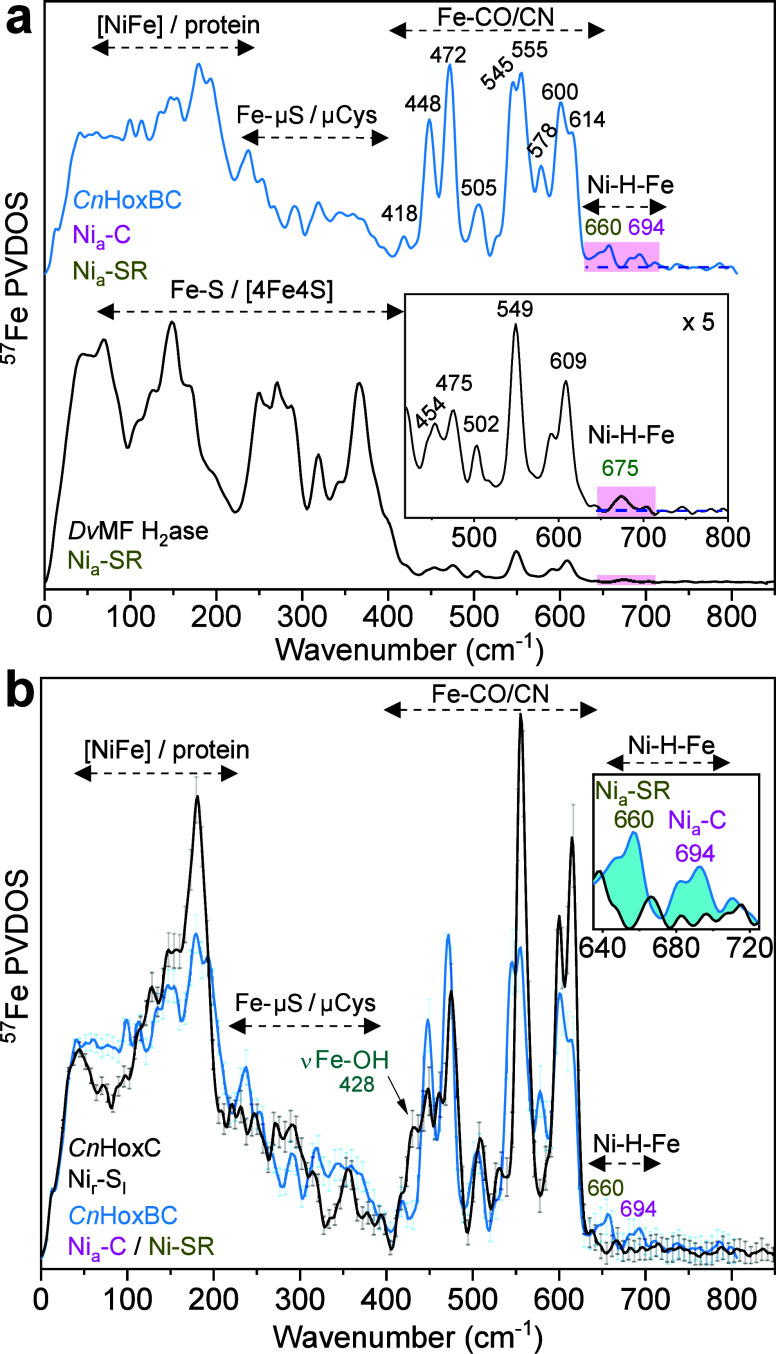
NRVS characterization of the assembled HoxBC complex. (a) HoxBC_red_ (*Cn*RH, upper trace; ∼60% Ni_a_-C, 40% Ni_a_-SR) and H_2_-reduced *Dv*MF (bottom trace; ∼80% Ni_a_-SR reproduced from ref 22. Available under a CC-BY 4.0 license. Copyright 2015 Springer Nature or Ogata, H., *et al*. Inset: 5× magnification of the Fe-CN/CO/H region, whose Ni–H–Fe part is highlighted in magenta. (b) Overlay of as-isolated HoxC (black) and HoxBC_red_ (light blue) showing successful subunit assembly. Inset: enlargement of hydride-associated signals (cyan). Spectral regions corresponding to vibrational modes of the [NiFe] active site are marked with dashed horizontal arrows, which are used consistently across all other figures.

Additional spectral features, primarily due to Fe–CN vibrational modes, occur at 418, 448, 472, and 505 cm^–1^.^19,23,26^ In line with our recent NRVS studies of purified [NiFe]-hydrogenase large subunits, we also resolved active site-related features in the low-frequency region of the spectrum that are usually masked by iron–sulfur cluster absorptions in uniformly ^57^Fe-labeled [NiFe]-hydrogenases (Figure 2a). Consistent with the findings of an earlier NRVS/DFT study,^26^ the isolated HoxC subunit occurred in the resting Ni_r_-S_I_ state (Figures 2b and S3), which contains a bridging OH^–^ ligand. In fact, the band at ca. 428 cm^–1^ in the HoxC spectrum was previously shown to contain large contributions of the bridging OH^–^ ligand in the form of an Fe–OH stretching vibration.^26^ The absence of this band in the HoxBC_red_ spectrum provides direct information on the assembly of the two hydrogenase subunits, which is associated with the removal of the OH^–^ ligand and the enrichment of catalytic intermediates.^3,26^ Strikingly, the spectrum of HoxBC_red_ contains two additional small bands centered at 660 and 694 cm^–1^ (Figure 2a, b) that are absent from the newly recorded spectrum of as-isolated HoxC (Figures 2b and S4). The two bands fall within the same spectral range as the previously detected bridging hydride wag modes (at ∼675 cm^–1^) for the Ni_a_-SR state of the *Dv*MF hydrogenase,^22^ and computed (∼694 cm^–1^) for the Ni_a_-C of *Cn*RH.^23^ Given that metal-bound hydride bands are sensitive to H/D isotope substitution,^22^ another HoxBC sample was prepared in D_2_/D_2_O (see methods).

The IR spectrum of deuterated HoxBC_red_ exhibited both the Ni_a_-C and the two Ni_a_-SR subforms (Figure 1, blue trace), with the Ni_a_-SR subforms slightly less enriched compared to the H_2_/H_2_O analog. Despite a moderately lower signal-to-noise ratio, the NRVS data of deuterated HoxBC_red_ exhibit no clear absorptions in the Fe–H spectral range (650–740 cm^–1^; Figures 3a and S5), consistent with the presence of a deuteride bridge in both Ni_a_-SR and Ni_a_-C. Consequently, all Fe–CO/CN bands of the deuterated sample display small redshifts (1–8 cm^–1^), and a distinct H/D-sensitive band at 578 cm^–1^ was identified in the H_2_/H_2_O-treated sample (Figure 3a, red rectangle), which was absent in the deuterated sample. Notably, the weak band at 694 cm^–1^, which we attribute to the Ni_a_-C state, is in agreement with recent DFT predictions,^23^ supporting its assignment to the Fe–H wagging mode of this intermediate. Given that the IR data for HoxBC_red_ (Figure 1) indicate significant amounts of two Ni_a_-SR subforms, the second NRVS band at about 660 cm^–1^, resolved without magnification of the spectra, can be tentatively assigned to Ni_a_-SR. The bridging hydride species are further supported by in situ photolysis experiments. Ni_a_-C, and more recently a Ni_a_-SR subform,^27^ have been shown to convert into Ni_a_-L species upon illumination at low temperatures. During this process, the hydride electrons are retained on nickel, generating a formal Ni^1+^ species, while the proton binds to a Ni-coordinating cysteine (Cys479 in *Cn*RH, Figure S1).^28–30^ Indeed, NRVS data collected on another HoxBC_red_ sample irradiated at 90 K show the disappearance of the Ni_a_-C/Ni_a_-SR hydride bands at 694 and 660 cm^–1^ (and the associated band at 578 cm^–1^),^23^ consistent with photolysis of both hydride species and the predominant formation of the Ni_a_-L2 intermediate (Figures 3b and S1b).^28^

**Figure 3:**
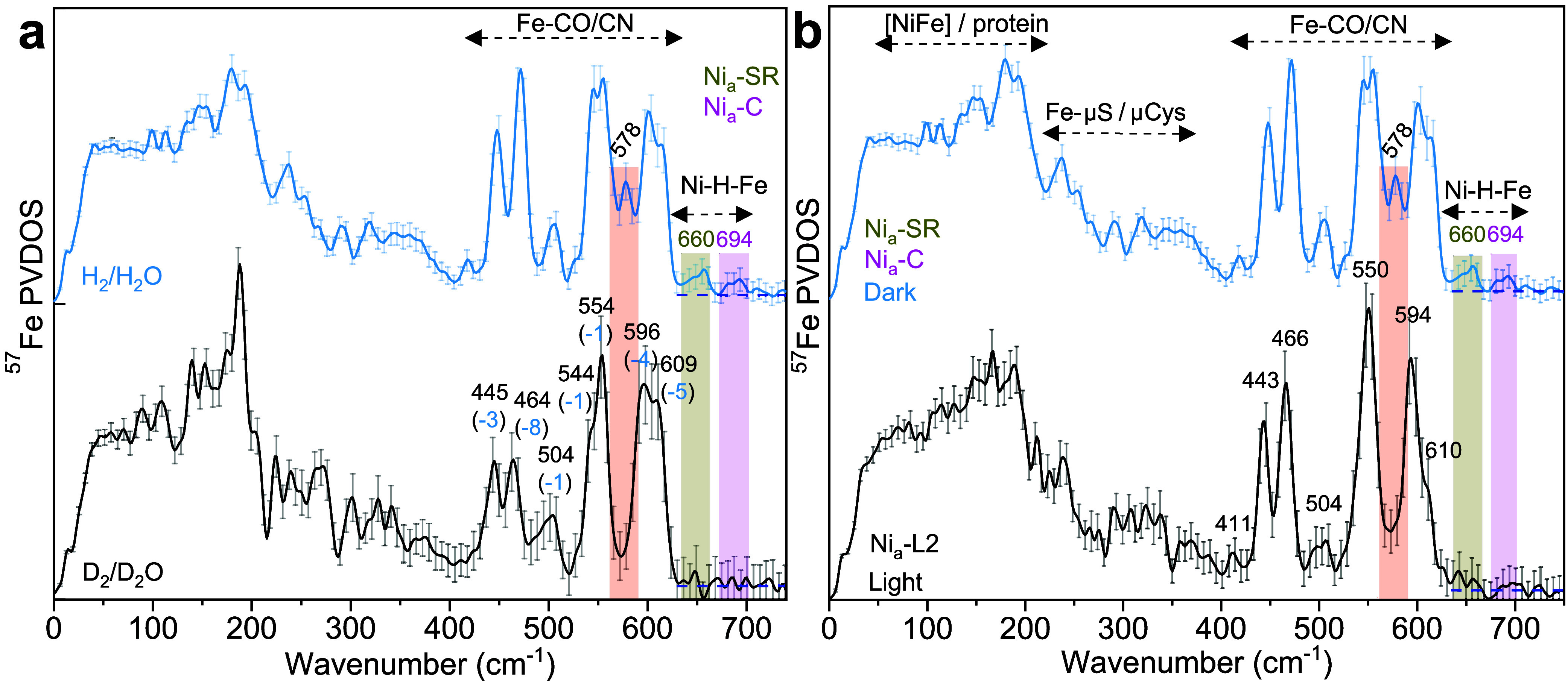
NRVS spectra of HoxBC_red_. (a) Spectra recorded in H_2_/H_2_O (light blue) and D_2_/D_2_O (black). (b) Spectrum after white-light illumination (black, mainly Ni_a_-L2). Fe–H–Ni-related bands of Ni_a_-SR and Ni_a_-C are highlighted in light red, olive yellow, and magenta bars. The average background level in the high-frequency region is indicated by dashed horizontal lines.

The simultaneous detection of two hydride species separated by a relatively large energy gap (Δν ≈ 34 cm^–1^, Figure 3a) is consistent with some structural differences, in particular a weaker (elongated) Fe–H bond in Ni_a_-SR. To estimate Fe–H bond lengths, we applied a Badger-type empirical correlation (derivation in the SI): *r* = *d* + (*r*_ref_ – *d*)­(ν_ref_/ν)^2/3^, where *r* is the equilibrium bond distance, ν the vibrational frequency, and *d* is the empirical Badger offset (0.85 Å for Fe–H^31^). Using the bridging hydride wagging mode of Ni_a_-SR in *Dv*MF (ν_ref_ = 675 cm^–1^) together with the corresponding Fe–H distance (1.78 Å) from the ultrahigh-resolution crystal structure (PDB: 4U9H) as a reference,^22,32^ we obtain semiquantitative estimates of ∼1.79 Å for Ni_a_-SR and ∼1.76 Å for Ni_a_-C in HoxBC, corresponding to a contraction of ∼0.03 Å. This small but significant change, which has not been reported for [NiFe]-hydrogenases to date, likely reflects the more electron-rich character of Ni_a_-SR (Ni^2+^) compared to Ni_a_-C (Ni^3+^), which can weaken the Fe–hydride interaction. Compared to biomimetic [NiFe] models containing a bridging hydride,^33,34^ for which only Ni_a_-SR mimics are available but no Ni_a_-C analogues,^35,36^ the active site of [NiFe]-hydrogenases is characterized by longer Fe–H bonds, slightly displaced toward Ni (d_Ni–H_ ≈ 1.58 Å in *Dv*MF hydrogenase). This subtle, yet functionally relevant structural difference likely contributes to the enzyme’s catalytic efficiency (Table S1). The displaced hydride may improve active site reactivity and facilitate more efficient proton–electron coupling, in contrast to the more symmetric hydride geometry observed in synthetic [NiFe] complexes.^37^ Lastly, comparison of the spectra of HoxBC in the Ni_a_-SR/Ni_a_-C, Ni_a_-L2, and Ni_a_-S states reveals clear similarities both in the Fe–CO/CN region, indicating preserved structural rigidity, and the low-frequency range (Figure 4, colored bars). The latter is dominated by torsional modes of the [NiFe] center (150–220 cm^–1^), which reflect internal motions of the [NiFe] coordination environment, and by cofactor displacements relative to the protein scaffold (50–150 cm^–1^), corresponding to collective, large-amplitude “soft” modes.^26^ Minor differences around ∼130 cm^–1^ likely reflect subtle local changes in the protein matrix—such as side-chain or hydrogen-bond reorientations—rather than major rearrangements of the active site.

**Figure 4:**
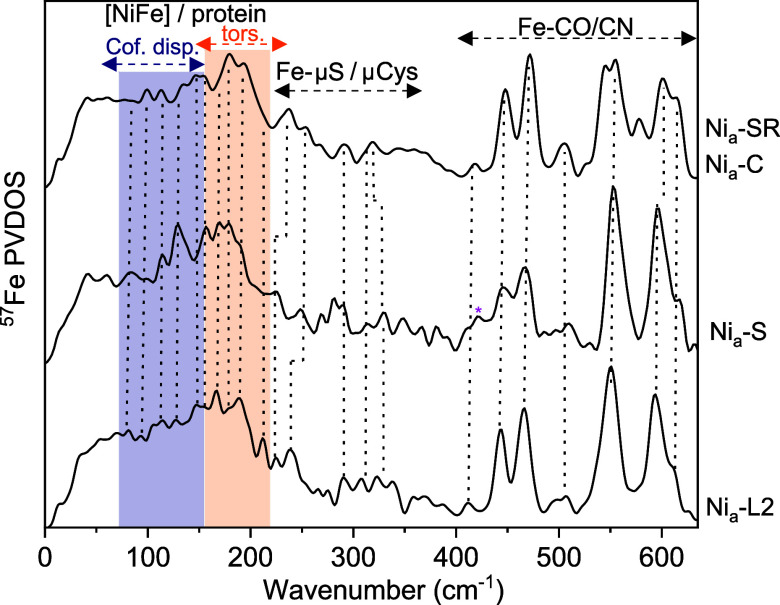
Full set of Fe–ligand vibrations of the [NiFe] site in HoxBC enriched in the Ni_a_-S, Ni_a_-L2, and Ni_a_-C/Ni_a_-SR intermediates. Low-frequency [NiFe]/protein modes comprising torsional (tors., orange)/breathing modes and cofactor displacements (cof. disp., navy) relative to the protein scaffold are indicated by dashed arrows * indicates remnants of Ni_r_-S_I_ (Figure 2b). Ni_a_-S data are reproduced from ref 24. Copyright 2020 American Chemical Society.

Our results are in excellent agreement with the recent crystallographic studies by Ash, Vincent, and co-workers,^38^ who reported minimal changes in the primary coordination sphere of the [NiFe]-hydrogenase Hyd-1 from *E. coli* during the catalytic cycle. In summary, we have, for the first time, resolved the full set of Fe–ligand vibrations of the [NiFe]-hydrogenase active site across all yet available catalytic intermediates. These results highlight that the catalytic site (i) maintains structural rigidity across all catalytically competent states, a feature likely essential for rapid electron and proton transfer during hydrogen turnover, and (ii) combines this rigidity with subtle variations in hydride coordination. These two factors are likely crucial for efficient catalysis and provides design principles for next-generation biomimetic [NiFe] complexes,^37^ underscoring that replicating these structural features is key to improving their performance.

## Supplementary Material



## Notes

Cultivation of bacteria and protein purification, Fe–H bond length estimation of the Ni_a_-SR and Ni_a_-C states of HoxBC using a Badger-type correlation, methods, supplementary figures and table, and supplementary references (PDF)
