# Supplementary material for: NRVS Spectroscopy Resolves Distinct Bridging Hydride Intermediates in [NiFe]-Hydrogenase
Source: J Am Chem Soc. 2025 Oct 31;147(45):41216–20. doi: 10.1021/jacs.5c15408 (PMC12616680; doi:10.1021/jacs.5c15408)
Supplement: Supplementary file 1 [file ja5c15408_si_001.pdf]

Supporting Information

NRVS Spectroscopy Resolves Distinct Bridging Hydride Intermediates in [NiFe]-Hydrogenase

Giorgio Caserta,[a]* Konstantin Laun,[a] Jean-Pierre Oudsen,[a] Ilya Sergueev,[b] Ingo Zebger,[a] and Oliver Lenz[a]

[a] Institut für Chemie, Technische Universität Berlin, Straße des 17. Juni 135, 10623 Berlin, Germany

[b] Deutsches Elektronen-Synchrotron, Notkestraße 85, 22607 Hamburg, Germany

Table of Contents

Methods

Cultivation of bacteria and protein purification

IR spectroscopy

Nuclear resonance vibrational spectroscopy

Fe–H bond length estimation of the Ni_a-SR and Ni_a-C states of HoxBC using a Badger-type correlation

Supplementary figures

Figure S1. Schematic representation of the proposed catalytic mechanism in [NiFe]-hydrogenases involving Ni_a-S, Ni_a-SR/'/'', Ni_a-C and Ni_a-L1/2/3 species.

Figure S2. IR spectra of the air-oxidized and H_2-reduced HoxBC complex.

Figure S3. IR absorbance spectrum of as-isolated HoxC.

Figure S4. IR spectra of the air-oxidized and H_2-reduced HoxBC complex**.**

Figure S5. Overlay of NRVS spectra of as-isolated HoxC (Ni_r-S_I) recorded at Petra III and SPring-8.

Table S1. Comparison of selected [NiFe]-hydrogenases and biomimetic NiFe complexes in terms of their k_{cat} values for both H_2 oxidation and H^+ reduction.

Supplementary references

Methods

Cultivation of bacteria and protein purification

Recombinant *C. necator* strain HF574 (pGE537) carrying the plasmid for overproduction of HoxC was cultivated in media containing ^{57}Fe as described previously.[1] When the bacterial cultures reached an optical density at 436 nm of 11-13, the cells were harvested by centrifugation (11,500 x *g*, 4 °C, 15 min), and the cell pellet was flash frozen in liquid nitrogen and stored at –80 °C until further use.
Heterologous HoxB production in *E. coli* was performed according to a previously reported procedure.[2] Cell pellets of recombinant strains were resuspended in lysis buffer (5 mL of buffer per g wet cell paste) consisting of 50 mM Tris-HCl, pH 8.0, 150 mM NaCl, protease inhibitor cocktail (cOmplete EDTA-free, Roche) and DNase I (Roche). The cells were subsequently disrupted in a French pressure cell (G. Heinemann Ultraschall and Labortechnik, Schwäbisch Gmünd, Germany) at 125 MPa. Crude extracts were ultracentrifuged for 40 min at 100,000 x *g* and 4 °C, and the resulting soluble extract was loaded onto a Strep-Tactin® high-capacity column (IBA, Göttingen, Germany). The column was washed with ten bed volumes of washing buffer (50 mM Tris-HCl, pH 8.0, 150 mM NaCl), and the proteins were eluted with 4 bed volumes of washing buffer containing 3 mM D-desthiobiotin. The eluted proteins were concentrated by ultrafiltration (4,000 x *g*, 4 °C) using Amicon Ultracel concentrators (Millipore) with a 30 kDa (HoxC) and 10 kDa (HoxB) cut-offs. The resulting protein solution was diluted 20-fold with washing buffer and again re-concentrated by ultrafiltration. The final concentrate was flash-frozen and stored in liquid nitrogen. The protein concentration was determined using a Pierce BCA Protein Assay kit (Thermo Scientific) using bovine serum albumin (BSA) as standard.
In vitro assembly of HoxC and HoxB followed established procedures.[2] The assembled HoxBC complex is characterized by a specific activity of ~6.0 $U \cdot mg^{-1}$ (TOF of ca. 8.6 s^{-1}), which was measured spectrophotometrically as H_2-mediated reduction of methylene blue. The resulting $HoxBC_{red}$ complex was further reduced with freshly prepared sodium dithionite (10 mM NaDT in 50 mM Tris-HCl, 150 mM NaCl, pH 8.0, 10 °C). For preparation of the deuterated analogue, both HoxB and HoxC were diluted 20-fold with deuterated buffers (50 mM Tris-HCl, 150 mM NaCl, lyophilized twice and dissolved in D_2O) and incubated with D_2 gas and NaDT inside an anaerobic work station. After 1 h, the sample was concentrated to ~0.8 mM. A small amount of precipitation was observed, but IR quality control of the supernatant confirmed the presence of both Ni_a-C and Ni_a-SR species. The sample was flash-frozen in an NRVS cell and stored in liquid nitrogen until beamtime.

IR spectroscopy

For IR measurements, 8-10 µl of the reconstituted and H_2/dithionite-treated (final concentration of NaDT is 10 mM in 50 mM Tris-HCl, 150 mM NaCl, pH 8.0 at 10 °C) $HoxBC_{red}$ samples were transferred into a homemade, gas-tight and temperature-controlled (10 °C) transmission cell equipped with two

sandwiched CaF_2 windows that are separated by a Teflon spacer (optical path length of 50 µm). Spectra with a resolution of 2 cm^{-1} were recorded by averaging 200 scans using a Tensor 27 Fourier-transform spectrometer (Bruker) equipped with a liquid nitrogen-cooled mercury-cadmium-telluride detector. The Bruker OPUS software 7.8 was used to acquire and analyze the data. A buffer spectrum was used as reference to calculate the corresponding absorbance spectra. OriginPro 2021 software was used to prepare figures.

Nuclear resonance vibrational spectroscopy (NRVS)

Freshly assembled and H_2/dithionite-treated HoxB-[^{57}Fe]C samples were concentrated to 1.0-1.2 mM and a volume of about 40 µl was filled into a Kapton tape-sealed copper cell, flash frozen and stored in liquid nitrogen until the start of beamtime measurements. NRVS measurements were conducted at Petra III P01 (Germany, http://petra3.desy.de) with fluxes of ~$6.4 \cdot 10^9$ photons/s, respectively, using 14.41 keV radiation (^{57}Fe). The experimental setup at P01 comprises a two-step monochromatization of the beam (energy resolution of 1.0 meV) and detection of the delayed nuclear fluorescence and the Kα fluorescence following nuclear excitation by avalanche photo diodes. Raw NRVS data were converted to single-phonon ^{57}Fe partial vibrational densities of states (PVDOS) using the PHOENIX software package (https://www.spectra.tools/).[3] The energy scales were calibrated with a [NEt_4][$^{57}FeCl_4$] sample, characterized by two prominent peaks at 378 cm^{-1} (asymmetric Fe–Cl stretching mode) and 139 cm^{-1} (Fe–Cl bending mode). The temperature of the samples was maintained at ca 12 K using a liquid He cryostat. The Stoke/anti-Stoke imbalance-derived real sample temperatures were 20-40 K. To emphasize the region of interest, sectional measurements of the spectral regions were performed. Each scan was divided into segments with different data collection times (second per point, s/pt). We used 3–4 s/pt for the region from –80 to 360 cm^{-1} and 8–9 s/pt for the region from 360 to 800 cm^{-1}. The detection of small Fe–H features required ~40 h of measurements in accordance with previous measurements on the *Dv*MF hydrogenase.[4,5] In situ hydride photolysis was carried out by irradiating frozen $HoxBC_{red}$ samples (loaded into NRVS cell) with a white-light source for ~1 h. During sample loading, the local temperature increased from ~12 K to ~110 K, a regime in which the Ni_a-L1 species is known to thermally relax to the more stable Ni_a-L2 state.[6] Consequently, the NRVS dataset (collected over ~18 h) is expected to predominantly contain contributions from the Ni_a-L2 species.

Fe–H bond length estimation of the Ni_a-SR and Ni_a-C states of HoxBC using a Badger-type correlation

Badger's equation

$$k = \frac{a}{(r-d)^3} \tag{1}$$

describes empirical relation between the bond force constant k and bond length r,[7] where a and d are empirical constants that establish the relation between vibrational frequency v and force constant according to the principle of the harmonic oscillator.

The relation between frequency v and the force constant k of a harmonic vibrator is

$$\nu = \frac{1}{2}\pi \cdot \sqrt{\frac{k}{\mu}} \qquad (2)$$

with μ being the reduced mass, which cancels itself out in ratios.

Substituting the Badger's equation into equation (2) gives

$$\nu \propto \sqrt{k} = \sqrt{a} \cdot \sqrt{\frac{1}{(r-d)^3}} \qquad (3)$$

or transformed

$$\sqrt{\frac{1}{(r-d)^3}} \propto \nu \Longrightarrow (r-d) \propto \sqrt[3]{\nu^{-2}} \qquad (4)$$

The introduction of the proportionality constant K leads to

$$r - d = K \sqrt[3]{\nu^{-2}} \qquad (5)$$

Since K is unknown, it can be eliminated by introducing a reference (r_{ref}, ν_{ref}), leading to

$$r_{ref} - d = K \sqrt[3]{\nu^{-2}{}_{ref}} \qquad (6)$$

The ratio of the equations (5) and (6) yields:

$$\frac{(r_{ref}-d)}{(r-d)} = \frac{\left(K \cdot \sqrt[3]{\nu^{-2}{}_{ref}}\right)}{\left(K \cdot \sqrt[3]{\nu^{-2}}\right)} \qquad (7)$$

Solving for r gives

$$r = d + \left(r_{ref} - d\right) \cdot \sqrt[3]{(\frac{\nu_{ref}}{\nu})^2} \qquad (8)$$

The constants a and d (and thus K) are determined empirically. Using a reference point [e.g., r_{ref}[8] =1.78, ν_{ref}[4]=675 cm^{-1}] eliminates the need to evaluate them explicitly. Because the wagging mode harbors both bending and stretching coordinates, these distances should be regarded as semi-quantitative estimates; however, (i) anchoring to the experimentally characterized *Dv*MF hydrogenase wagging helps minimize systematic errors, and (ii) the observed difference between Ni_a-SR and Ni_a-C exceeds the combined uncertainties from mode mixing and anharmonicity, consistent with a genuine structural change.

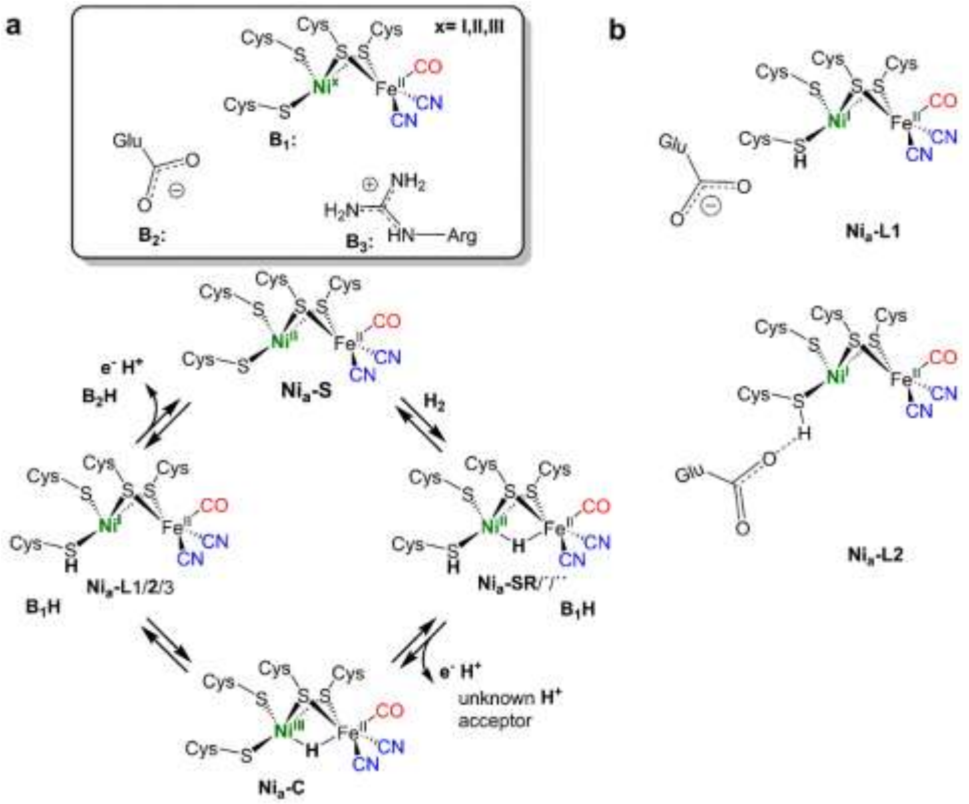

Fig. S1. (**a**) Schematic representation of the proposed catalytic mechanism of [NiFe]-hydrogenases involving the intermediates Ni_a-S, Ni_a-SR, Ni_a-C, and Ni_a-L.[9–12] During catalysis, only the nickel center undergoes redox transitions, whereas the iron remains in a low-spin Fe(II) configuration throughout the catalytic cycle. Three amino acid residues located near the active site have been discussed as potential proton acceptors: a Ni-bound terminal cysteine (Cys479 in *Cn*RH, B_1:), a nearby glutamate (Glu13 in *Cn*RH, B_2:), and an arginine (Arg411 in *Cn*RH, B_3:). These residues are shown in the top panel in their protonated/deprotonated states under physiological conditions. Ni_a-SR and Ni_a-L intermediates comprise three sub-forms, two of which (Ni_a-L2 and Ni_a-SR, bold) have been extensively characterized. Recent IR studies on *Cn*RH[6] and *Dv*MF[13] hydrogenases indicate that the Ni-bound cysteine (B_1, Cys479 in *Cn*RH) is protonated in the Ni_a-L1/2 intermediates of the cycle (**b**), with Ni_a-L2 further stabilized by a hydrogen bond to the deprotonated Glu (B_2:, Glu13 in *Cn*RH). Although all previously reported [NiFe]-hydrogenase structures of catalytic intermediates show minimal or no changes in both the primary and outer coordination spheres of the [NiFe] site, two conformations of the conserved glutamate were identified in *Cn*RH using low-temperature IR difference spectroscopy[6] and were recently resolved structurally in two Ni_a-L subforms of Hyd-1 and Hyd-2 from *E. coli*.[14] In addition, an ultra-high-resolution structure of *Dv*MF hydrogenase in the Ni_a-SR state revealed that the analogous Ni-bound cysteine is also protonated in this intermediate.[8] Spectroscopic and site-directed mutagenesis studies on the soluble [NiFe]-hydrogenase from *Pyrococcus furiosus* (*Pf*SH1) established that the conserved glutamate functions as the proton acceptor during the Ni_a-L → Ni_a-S conversion,[15] whereas the identity of the proton acceptor in the Ni_a-SR → Ni_a-C transition remains unresolved.

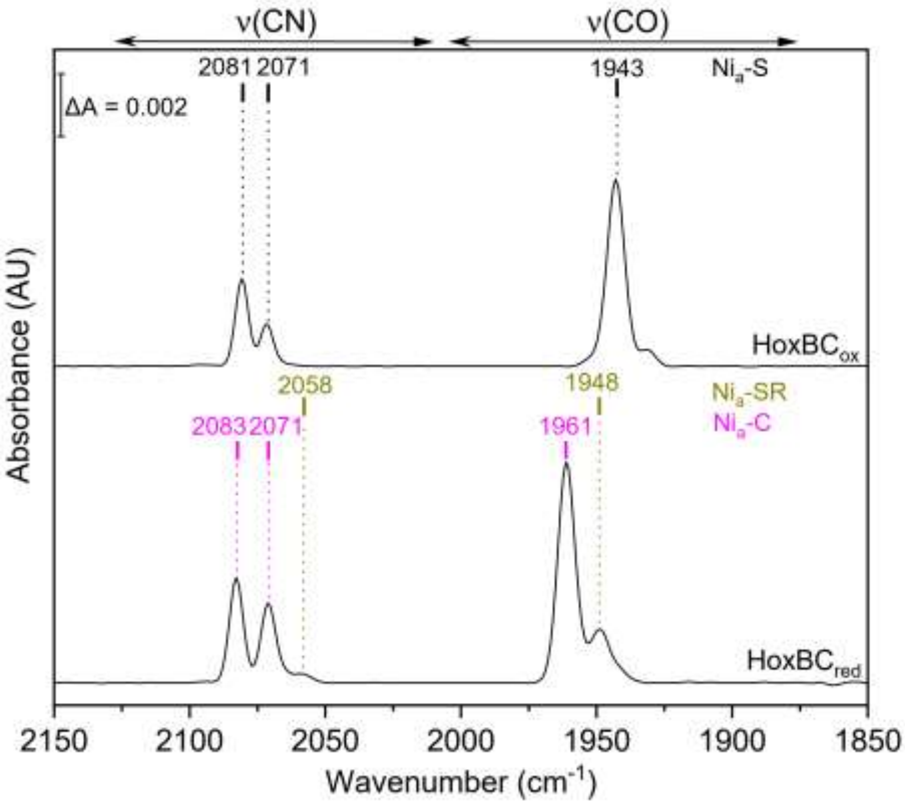

Fig. S2. IR spectra of the air-oxidized and H_2-reduced HoxBC complex (T=283 K). The IR bands, related to the stretching vibrations of the CO and CN^- ligands bound to the Fe of the [NiFe]-hydrogenase active site, are sensitive to redox structural changes of the catalytic center. Color code: Ni_a-C, magenta; Ni_a-SR, olive yellow; Ni_a-S, black. CO/CN absorptions are labeled with the corresponding wavenumbers.

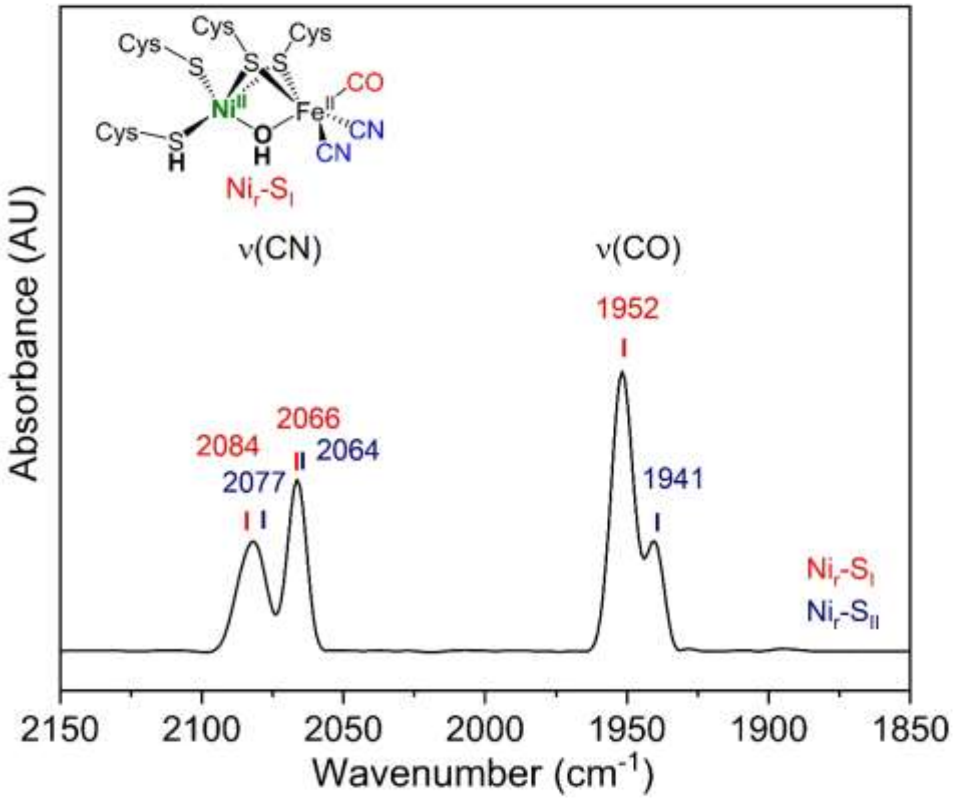

Fig. S3. IR absorbance spectrum of as-isolated HoxC labeled with ^{57}Fe recorded at pH 8.0 (T=283 K). The positions of the IR bands are related to the stretching vibrations of the CO and CN ligands of the $NiFe(CN)_2CO$ site and reflect contributions of the Ni_r–S_I (red) and Ni_r–S_{II} (blue) resting states, respectively.[1,16] CO/CN absorptions are labeled with the corresponding wavenumbers. A sketch of the Ni_r-S_I structure containing a bridging hydroxy ligand is shown in the upper left corner.

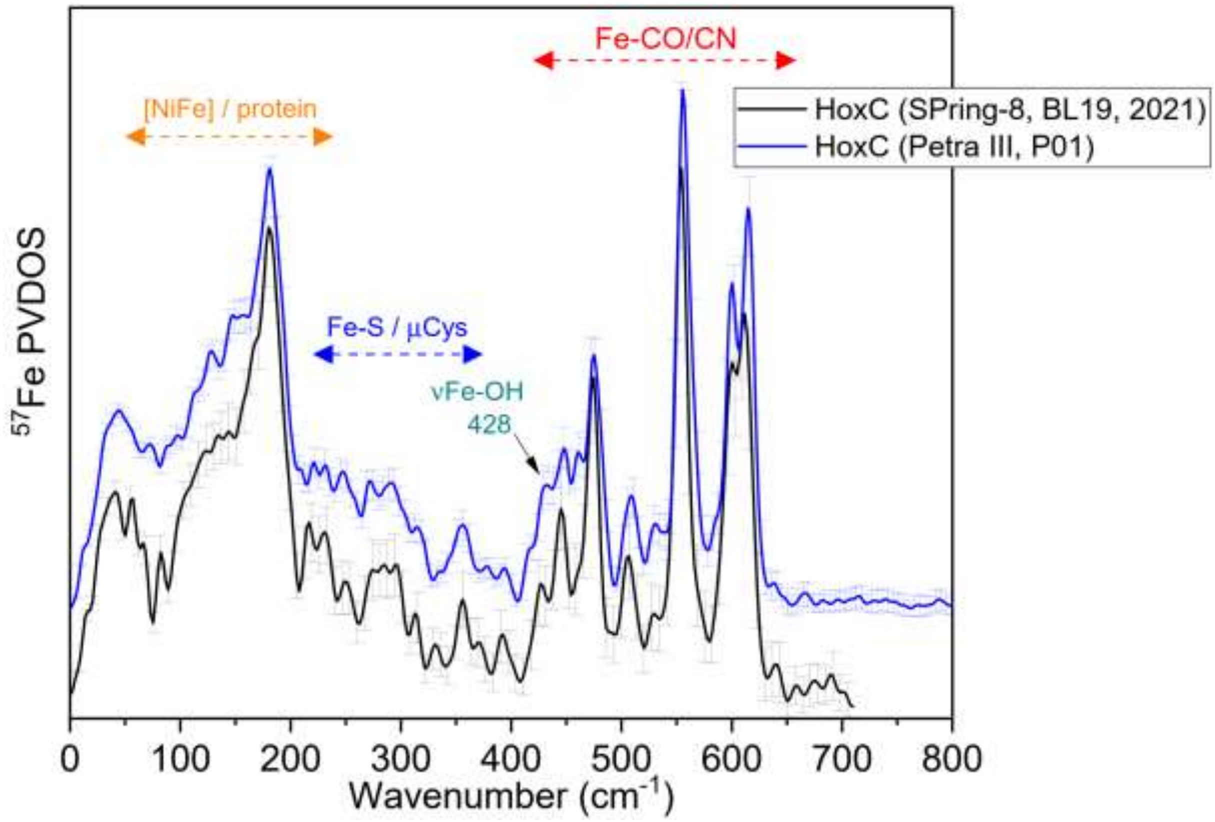

Fig. S4. Overlay of NRVS spectra of as-isolated HoxC (Ni_r-S_I) recorded at Petra III (blue trace) and SPring-8 (black trace[16]). Dashed arrows indicate spectral regions: red, Fe–CO/CN; orange, [NiFe]/protein modes; blue, Fe–S (bridging cysteines). The absorption band at ca. 428 cm^{-1} (dark cyan) contains large contributions of the bridging OH^- ligand in the form of an Fe−OH stretching vibration. A new HoxC sample (ca 1.2 mM, average of 24 scans) was measured to reduce error bars in the 600–800 cm^{-1} region, which is characteristic of bridging hydride wagging modes and facilitates the assignment of Fe–H bands in $HoxBC_{red}$ at 660 and 694 cm^{-1} (**Fig. 2b**).

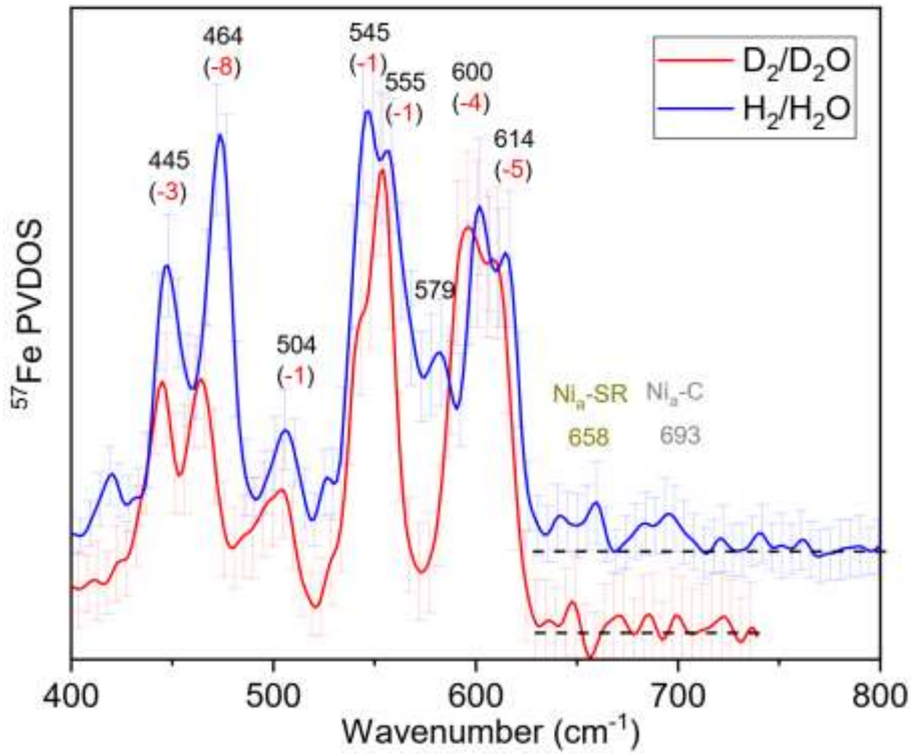

Fig. S5. NRVS spectra of H_2/H_2O-treated HoxBC$_{red}$ (blue trace, ~1.2 mM, average of 15 scans) and D_2/D_2O-treated HoxBC$_{red}$ (red trace, ~0.8 mM, average of 20 scans) in the 400–740 cm^{-1} region, where absorptions associated with Fe–CO/CN and Ni–H–Fe vibrations of the active site are observed. Relevant bands are labeled with their corresponding wavenumbers. By reducing the number of scans for the H_2/H_2O-treated sample, the error bars (thin vertical lines) remain comparable in size to those of the D_2/D_2O-treated sample across the entire spectral range. In the high-frequency region, the H_2/H_2O spectrum shows distinct bridging hydride bands for the Ni$_a$-C and Ni$_a$-SR species, which are absent in the deuterated sample due to H/D substitution (further corroborated by the disappearance of the 579 cm^{-1} band in line with data on *Dv*MF hydrogenase and lyophilized *Cn*RH).[4,17] The spectrum of the D_2/D_2O-treated sample exhibits a signal-to-noise ratio that is reduced by a factor of approximately two compared to the corresponding H_2/H_2O sample (**Fig. 3a**), as evidenced by increased baseline fluctuations. Extended accumulation times for the D_2/D_2O-treated sample did not improve the statistics, primarily due to limited sample stability under the experimental conditions. The average background level in the high-frequency range is indicated by dashed horizontal black lines.

Table S1. Comparison of selected [NiFe]-hydrogenases and biomimetic NiFe complexes in terms of their k_{cat} values for both H_2 oxidation and H^+ reduction.

H_2 oxidation		H^+ reduction	
Sample	k_{cat}	Sample	k_{cat}
[NiFe]-hydrogenase from *Allochromatium vinosum*	~ 6000 s^{-1} [18]	NAD^+-reducing [NiFe]-hydrogenase from *Synechocystis* PCC 6803	~1300 s^{-1} [19]
O_2 tolerant NAD^+-reducing [NiFe]-hydrogenase from *C. necator*	340 s^{-1} [20]	[NiFeSe]-hydrogenase from *D. vulgaris*	~10000 s^{-1} [21]
[NiFe]-hydrogenase from *Citrobacter* sp S-77	~ 900000 s^{-1} [22]	[L^{OMe}NiFeCp]	~16000 s^{-1} [23]
NiFe complex 1	1 TON*[24]	[LNiFeCp]	~ 200 s^{-1} under mildly acidic aqueous conditions [25]

*Despite advancements in synthetic chemistry, no [NiFe] biomimetic complex has been identified that can catalyze H_2 oxidation with measurable turnover rates. An exception are Dubois's bioinspired Ni complexes, e.g., $[Ni(P^{Cy}{}_2N^{Arg}{}_2)_2]^{8+}$ with *a* k_{cat} of ~ 210 s^{-1}.[26,27]

Supplementary references

(1) Caserta, G.; Lorent, C.; Ciaccafava, A.; Keck, M.; Breglia, R.; Greco, C.; Limberg, C.; Hildebrandt, P.; Cramer, S. P.; Zebger, I.; Lenz, O. The Large Subunit of the Regulatory [NiFe]-Hydrogenase from *Ralstonia Eutropha* – a Minimal Hydrogenase? *Chem. Sci.* **2020**, *11* (21), 5453–5465. https://doi.org/10.1039/D0SC01369B.

(2) Caserta, G.; Lorent, C.; Pelmenschikov, V.; Schoknecht, J.; Yoda, Y.; Hildebrandt, P.; Cramer, S. P.; Zebger, I.; Lenz, O. *In Vitro* Assembly as a Tool to Investigate Catalytic Intermediates of [NiFe]-Hydrogenase. *ACS Catal.* **2020**, *10* (23), 13890–13894. https://doi.org/10.1021/acscatal.0c04079.

(3) Gee, L. B.; Wang, H.; Cramer, S. P. NRVS for Fe in Biology: Experiment and Basic Interpretation. In *Methods in Enzymology*; Elsevier, 2018; Vol. 599, pp 409–425. https://doi.org/10.1016/bs.mie.2017.11.002.

(4) Ogata, H.; Krämer, T.; Wang, H.; Schilter, D.; Pelmenschikov, V.; Van Gastel, M.; Neese, F.; Rauchfuss, T. B.; Gee, L. B.; Scott, A. D.; Yoda, Y.; Tanaka, Y.; Lubitz, W.; Cramer, S. P. Hydride Bridge in [NiFe]-Hydrogenase Observed by Nuclear Resonance Vibrational Spectroscopy. *Nat. Commun.* **2015**, *6* (1), 7890. https://doi.org/10.1038/ncomms8890.

(5) Wang, H.; Yoda, Y.; Ogata, H.; Tanaka, Y.; Lubitz, W. A Strenuous Experimental Journey Searching for Spectroscopic Evidence of a Bridging Nickel–Iron–Hydride in [NiFe] Hydrogenase. *J. Synchrotron Radiat.* **2015**, *22* (6), 1334–1344. https://doi.org/10.1107/S1600577515017816.

(6) T. Waffo, A. F.; Lorent, C.; Katz, S.; Schoknecht, J.; Lenz, O.; Zebger, I.; Caserta, G. Structural Determinants of the Catalytic Ni_a -L Intermediate of [NiFe]-Hydrogenase. *J. Am. Chem. Soc.* **2023**, *145* (25), 13674–13685. https://doi.org/10.1021/jacs.3c01625.

(7) Badger, R. M. The Relation Between the Internuclear Distances and Force Constants of Molecules and Its Application to Polyatomic Molecules. *J. Chem. Phys.* **1935**, *3* (11), 710–714. https://doi.org/10.1063/1.1749581.

(8) Ogata, H.; Nishikawa, K.; Lubitz, W. Hydrogens Detected by Subatomic Resolution Protein Crystallography in a [NiFe] Hydrogenase. *Nature* **2015**, *520* (7548), 571–574. https://doi.org/10.1038/nature14110.
(9) Lubitz, W.; Ogata, H.; Rüdiger, O.; Reijerse, E. Hydrogenases. *Chem. Rev.* **2014**, *114* (8), 4081–4148. https://doi.org/10.1021/cr4005814.
(10) Ash, P. A.; Hidalgo, R.; Vincent, K. A. Proton Transfer in the Catalytic Cycle of [NiFe] Hydrogenases: Insight from Vibrational Spectroscopy. *ACS Catal.* **2017**, *7* (4), 2471–2485. https://doi.org/10.1021/acscatal.6b03182.
(11) Greene, B. L.; Vansuch, G. E.; Chica, B. C.; Adams, M. W. W.; Dyer, R. B. Applications of Photogating and Time Resolved Spectroscopy to Mechanistic Studies of Hydrogenases. *Acc. Chem. Res.* **2017**, *50* (11), 2718–2726. https://doi.org/10.1021/acs.accounts.7b00356.
(12) Tai, H.; Hirota, S.; Stripp, S. T. Proton Transfer Mechanisms in Bimetallic Hydrogenases. *Acc. Chem. Res.* **2021**, *54* (1), 232–241. https://doi.org/10.1021/acs.accounts.0c00651.
(13) Tai, H.; Nishikawa, K.; Higuchi, Y.; Mao, Z.; Hirota, S. Cysteine SH and Glutamate COOH Contributions to [NiFe] Hydrogenase Proton Transfer Revealed by Highly Sensitive FTIR Spectroscopy. *Angew. Chem. Int. Ed.* **2019**, *58* (38), 13285–13290. https://doi.org/10.1002/anie.201904472.
(14) Carr, S. B.; Li, W.; Wong, K. L.; Evans, R. M.; Kendall-Price, S. E. T.; Vincent, K. A.; Ash, P. A. Glutamate "Flick" Enables Proton Tunneling during Fast Redox Biocatalysis. *ChemRxiv.* 2024-05-06, https://doi.org/10.26434/chemrxiv-2024-mn36l.
(15) Greene, B. L.; Vansuch, G. E.; Wu, C.-H.; Adams, M. W. W.; Dyer, R. B. Glutamate Gated Proton-Coupled Electron Transfer Activity of a [NiFe]-Hydrogenase. *J. Am. Chem. Soc.* **2016**, *138* (39), 13013–13021. https://doi.org/10.1021/jacs.6b07789.
(16) Caserta, G.; Pelmenschikov, V.; Lorent, C.; Tadjoung Waffo, A. F.; Katz, S.; Lauterbach, L.; Schoknecht, J.; Wang, H.; Yoda, Y.; Tamasaku, K.; Kaupp, M.; Hildebrandt, P.; Lenz, O.; Cramer, S. P.; Zebger, I. Hydroxy-Bridged Resting States of a [NiFe]-Hydrogenase Unraveled by Cryogenic Vibrational Spectroscopy and DFT Computations. *Chem. Sci.* **2021**, *12* (6), 2189–2197. https://doi.org/10.1039/D0SC05022A.
(17) Lorent, C.; Pelmenschikov, V.; Frielingsdorf, S.; Schoknecht, J.; Caserta, G.; Yoda, Y.; Wang, H.; Tamasaku, K.; Lenz, O.; Cramer, S. P.; Horch, M.; Lauterbach, L.; Zebger, I. Exploring Structure and Function of Redox Intermediates in [NiFe]-Hydrogenases by an Advanced Experimental Approach for Solvated, Lyophilized and Crystallized Metalloenzymes. *Angew. Chem. Int. Ed.* **2021**, *60* (29), 15854–15862. https://doi.org/10.1002/anie.202100451.
(18) Jones, A. K.; Sillery, E.; Albracht, S. P. J.; Armstrong, F. A. Direct Comparison of the Electrocatalytic Oxidation of Hydrogen by an Enzyme and a Platinum catalyst *Chem. Commun.* **2002**, 866–867. https://doi.org/10.1039/b201337a.
(19) Gutekunst, K.; Hoffmann, D.; Westernströer, U.; Schulz, R.; Garbe-Schönberg, D.; Appel, J. In-Vivo Turnover Frequency of the Cyanobacterial NiFe-Hydrogenase during Photohydrogen Production Outperforms in-Vitro Systems. *Sci. Rep.* **2018**, *8* (1), 6083. https://doi.org/10.1038/s41598-018-24430-y.
(20) Lauterbach, L.; Lenz, O. Catalytic Production of Hydrogen Peroxide and Water by Oxygen-Tolerant [NiFe]-Hydrogenase during H_2 Cycling in the Presence of O_2. *J. Am. Chem. Soc.* **2013**, *135* (47), 17897–17905. https://doi.org/10.1021/ja408420d.
(21) Marques, M. C.; Tapia, C.; Gutiérrez-Sanz, O.; Ramos, A. R.; Keller, K. L.; Wall, J. D.; De Lacey, A. L.; Matias, P. M.; Pereira, I. A. C. The Direct Role of Selenocysteine in [NiFeSe] Hydrogenase Maturation and Catalysis. *Nat. Chem. Biol.* **2017**, *13* (5), 544–550. https://doi.org/10.1038/nchembio.2335.
(22) Matsumoto, T.; Eguchi, S.; Nakai, H.; Hibino, T.; Yoon, K.; Ogo, S. [NiFe]Hydrogenase from *Citrobacter* Sp. S-77 Surpasses Platinum as an Electrode for H_2 Oxidation Reaction. *Angew. Chem. Int. Ed.* **2014**, *53* (34), 8895–8898. https://doi.org/10.1002/anie.201404701.
(23) Lalaoui, N.; Suarez-Antuna, I.; Arjunan, S.; Curtil, M.; Ioannou, P.-C.; Molton, F.; Chavant, P. Y.; Philouze, C.; Milet, A.; Maldivi, P.; Duboc, C. Tuning the Electronic and Molecular Structures of

Bioinspired Heterodinuclear NiFe Catalyst for Enhanced Catalytic H_2 Evolution. *ACS Org. Inorg. Au* **2025**, *5* (4), 230–237. https://doi.org/10.1021/acsorginorgau.5c00019.

(24) Ogo, S.; Ichikawa, K.; Kishima, T.; Matsumoto, T.; Nakai, H.; Kusaka, K.; Ohhara, T. A Functional [NiFe]Hydrogenase Mimic That Catalyzes Electron and Hydride Transfer from H_2. *Science* **2013**, *339* (6120), 682–684. https://doi.org/10.1126/science.1231345.

(25) Ahmed, M. E.; Chattopadhyay, S.; Wang, L.; Brazzolotto, D.; Pramanik, D.; Aldakov, D.; Fize, J.; Morozan, A.; Gennari, M.; Duboc, C.; Dey, A.; Artero, V. Hydrogen Evolution from Aqueous Solutions Mediated by a Heterogenized [NiFe]-Hydrogenase Model: Low pH Enables Catalysis through an Enzyme-Relevant Mechanism. *Angew. Chem. Int. Ed.* **2018**, *57* (49), 16001–16004. https://doi.org/10.1002/anie.201808215.

(26) Dutta, A.; Roberts, J. A. S.; Shaw, W. J. Arginine-Containing Ligands Enhance H_2 Oxidation Catalyst Performance. *Angew. Chem. Int. Ed.* **2014**, *53* (25), 6487–6491. https://doi.org/10.1002/anie.201402304.

(27) Caserta, G.; Roy, S.; Atta, M.; Artero, V.; Fontecave, M. Artificial Hydrogenases: Biohybrid and Supramolecular Systems for Catalytic Hydrogen Production or Uptake. *Curr. Opin. Chem. Biol.* **2015**, *25*, 36–47. https://doi.org/10.1016/j.cbpa.2014.12.018.
